# Transient receptor potential vanilloid type 4 is expressed in vasopressinergic neurons within the magnocellular subdivision of the rat paraventricular nucleus of the hypothalamus

**DOI:** 10.1002/cne.24514

**Published:** 2018-11-06

**Authors:** F.C. Shenton, S. Pyner

**Affiliations:** ^1^ Department of Biosciences Durham University Durham UK

**Keywords:** osmolality. RRID:AB_10675981, oxytocin, preautonomic neurons, PVN, RRID:AB_142018, RRID:AB_2534095, RRID:AB_518526, RRID:AB_518680, sympathetic output, TRPV4 ion channel, vasopressin

## Abstract

Changes in plasma osmolality can drive changes in the output from brain centres known to control cardiovascular homeostasis, such as the paraventricular nucleus of the hypothalamus (PVN). Within the PVN hypotonicity reduces the firing rate of parvocellular neurons, a neuronal pool known to be involved in modulating sympathetic vasomotor tone. Also present in the PVN is the transient receptor potential vanilloid type 4 (TRPV4) ion channel. Activation of TRPV4 within the PVN mimics the reduction in firing rate of the parvocellular neurons but it is unknown if these neurons express the channel. We used neuronal tracing and immunohistochemistry to investigate which neurons expressed the TRPV4 ion channel protein and its relationship with neurons known to play a role in plasma volume regulation. Spinally projecting preautonomic neurons within the PVN were labelled after spinal cord injection of FluoroGold (FG). This was followed by immunolabelling with anti‐TRPV4 antibody in combination with either anti‐oxytocin (OXT) or anti‐vasopressin (AVP). The TRPV4 ion channel was expressed on 63% of the vasopressinergic magnocellular neurosecretory cells found predominantly within the posterior magnocellular division of the PVN. Oxytocinergic neurons and FG labelled preautonomic neurons were present in the same location, but were distinct from the TRPV4/vasopressin expressing neurons. Vasopressinergic neurons within the supraoptic nucleus (SON) were also found to express TRPV4 and the fibres extending between the SON and PVN. In conclusion within the PVN, TRPV4 is well placed to respond to changes in osmolality by regulating vasopressin secretion, which in turn influences sympathetic output via preautonomic neurons.

## 1 INTRODUCTION

In mammals, plasma osmolality is precisely regulated despite the daily variation in water and sodium intake (Bourque, 2008). The paraventricular nucleus of the hypothalamus integrates behavioural, cardiovascular and neuroendocrine homeostatic responses (Swanson & Kuypers, 1980; Swanson & Sawchenko, 1983; Guyenet, 2006). An increase in plasma osmolality activates the PVN to increase arginine vasopressin (AVP) release and sympathetic nerve activity to end organs such as the kidney and heart (Stocker, Hunwick, & Toney, 2005; Antunes, Yao, Pickering, Murphy, & Paton, 2006). This is dependent upon a neural pathway comprising osmoreceptors located in specific forebrain regions that lack the blood brain barrier. Increasing plasma osmolality stimulates the subfornical organ, the organ vasculosum of the lamina terminalis and median preoptic, which all send excitatory projections to the PVN. The multi‐tasking role of the PVN is made possible by bringing together within the nucleus different cell groups with distinct neuro‐hormonal functions (Swanson & Sawchenko, 1983). Two morphologically distinct cell groups have been described: magnocellular neurosecretory cells (MNCs) that synthesise and release the peptide hormones AVP and oxytocin (OXT) and project exclusively to the posterior pituitary; and parvocellular neurons, that are further subdivided into two groups: one which releases corticortropin‐releasing factor to evoke the release of adrenocorticotropic hormone from the anterior pituitary (Antoni, 1993) and another group, the preautonomic neurons that influences autonomic function via projections to the brain stem and spinal cord (Pyner & Coote, 1999, 2000). The spinally projecting preautonomic neurons have also been categorised as intermediate and named mediocellular (Kiss, Martos, & Palkovits, 1991). The preautonomic neurons influence blood pressure, heart rate and sympathetic nerve activity, including renal sympathetic nerve activity.

The transient receptor potential vanilloid type 4 channel (TRPV4) is a non‐selective cation channel transducing physical stress, for example, osmotic cell swelling or mechanical stress into intracellular Ca^2+^−dependent signalling (Sharif‐Naeini, Ciura, Zhang, & Bourque, 2008). More recently, it has been demonstrated that TRPV4 is activated by increased cell volume irrespective of the molecular mechanism underlying cell swelling and thus the channel is suggested to function as a volume‐sensor, rather than (or as well as) an osmo‐sensor (Toft‐Bertelsen, Krizaj, & MacAulay, 2017).

The TRPV4 channel may be involved in systemic osmoregulation and there is some evidence to support a physiological role for TRPV4 in the hypothalamic osmosensing nuclei (Liedtke & Friedman, 2003; Carreño, Ji, & Cunningham, 2009). The TRPV4 channel is expressed in the PVN and SON where it is co‐localised with AVP containing cells (Carreño, Ji, & Cunningham, 2009). Functionally, TRPV4 and calcium activated potassium (K_Ca_) ion channels have been shown to couple as osmosensors in the PVN in mouse brain slices and rat isolated PVN neurons (Feetham, Nunn, Lewis, Dart, & Barrett‐Jolley, 2015b). Again in mice, intracerebroventricular administration of hypotonic artificial cerebrospinal fluid decreases blood pressure but not heart rate and inhibition of the TRPV4 ion channel attenuated this effect (Feetham, Nunn, & Barrett‐Jolley, 2015a). While these studies demonstrate a functional role for TRPV4 in osmosensing within the PVN, they do not establish which neurons express the channel, leaving open the question of the neuronal mechanism underlying these observations. Here we have used retrograde labelling of spinally projecting preautonomic neurons in combination with immunohistochemistry for TRPV4, AVP and OXT to determine where TRPV4 protein is expressed and its relationship to cell groups involved in osmosensing and cardiovascular homeostasis within the PVN.

## 2 MATERIALS and METHODS

### 2.1 Ethical Approval

All experiments were approved by the Local Ethics Committee of Durham University and performed in accordance with UK Animals (Scientific Procedures) Act, 1986 and the European Commission Directive 86/609/EEC (European Convention for the Protection of Vertebrate Animals used for Experimental and Other Scientific Purposes). All surgical procedures were carried out on anaesthetised animals that minimised suffering with the minimum number of animals used. Animal were killed with an overdose of sodium pentobarbital (60 mg/kg) at the termination of the experiment.

### 2.2 Injection of retrograde tracers

Six male wistar rats were anaesthetised intraperitoneally with medetomidine 0.25 mL/100 g and ketamine 0.06 mL/100 g prior to spinal cord injection of the retrograde tracer FluoroGold (FG; Fluorochrome –Denver, Colorado, USA LLC). The FG (2% in 0.9% saline) was pressure injected into the left intermediolateral region of the spinal cord at the T2 level (Watkins, Cork, & Pyner, 2009). Following injection, analgesia was administered (0.01 mL/100 g buprenorphine) and the animals recovered for 7–10 days with *ad libitum* food and water.

### 2.3 Perfusion‐Fixation

After the recovery period, animals were terminally anaesthetised and perfused with heparinised saline followed by 4% paraformaldehyde in 0.1 M phosphate buffer (PB; pH 7.4). Brains and spinal cord were removed, post fixed overnight at 4 °C and then transferred to 30% sucrose‐phosphate buffer (4 °C) until sectioned.

### 2.4 Immunohistochemistry

Immunohistochemistry was carried out on free floating sections cut on a freezing microtome at 40 μm. Transverse sections of PVN were collected at the levels containing centres engaged in cardiovascular control (Swanson & Sawchenko, 1983; Pyner & Coote, 1999) and longitudinal sections of spinal cord (100 μm) were used to confirm the location of the injection site within the intermediolateral cell column (Swanson & Sawchenko, 1983; Pyner & Coote, 1999). Nonspecific binding sites were blocked with 10% normal goat serum (NGS; Abcam Cambridge CB4 0FL, UK, Ab7481)‐0.1% Triton‐X‐100 (TX) in PB for 45 minutes, rinsed in PB (1 x 10 mins) then incubated in rabbit anti‐TRPV4 (1:400 in 1% NGS‐0.1% TX in PB; Abcam 94,868 lot GR276084, RRIDAB_10675981) overnight at 4 °C. Four animals underwent double labelling for anti‐TRPV4 combined with either guinea pig anti‐oxytocin (1:1000; BMA Biomedicals, CH‐4302 Augst, Switzerland, T‐5021.0050, RRID:AB_518526) or guinea pig anti‐(Arg 8) vasopressin (1:800; BMA Biomedicals, T‐5048.0050, RRID:AB_518680). After washing (x 3 in PB) the secondary antibody, either Alexafluor 594 goat anti‐rabbit (1:200; ThermoFisher, UK, A‐11037, RRID:AB_2534095) alone or together with Alexafluor 488 anti‐guinea pig (1:200; ThermoFisher, UK, A‐11073, RRID:AB_142018) for double labelled sections, was applied for 2 hours at room temperature. Finally, the sections were washed as before and mounted onto gelatinised slides. After air drying overnight, sections were dehydrated through a series of alcohols, cleared in xylene and then mounted under DPX.

### 2.5 Confocal Microscopy

Sections were examined using a Zeiss 880 Laser Scanning Confocal Microscope. Images were captured using Zen 2.1 SP2 (black; version 13.0.2.518). Frame mode acquisition was utilised to capture FluoroGold (excitation 405 nm, emission 530–600 nm), Alexafluor 488 (excitation 488, nm emission 494–600 nm) and Alexaflour 594 (excitation 594 nm, emission 604–735 nm). Overview images were captured using x20 objective (NA 0.8) in tile scan mode to generate the large field of view required and z stacks as required. Regions of interest were subsequently imaged with either x40 or x63 oil objectives (NA 1.3 and 1.4 respectively). Raw images were processed using Zen (blue edition) software and final images were imported into Adobe Photoshop (CS4 extended v. 11.02) to create annotated figures.

### 2.6 Cell Counts

Cell counts were generated using a cell counter plugin in the Java‐based image processing program ImageJ (https://imagej.nih.gov/ij/, 1997–2016.). FluoroGold labelled neurons were counted in consecutive sections throughout the rostrocaudal extent of the PVN, from approximately Bregma −1.40 to −2.12, ipsilateral to the spinal cord injection site. Abercrombie's correction for double counting errors was applied to these counts (Abercrombie, 1946). In four animals alternate sections were labelled with anti‐TRPV4 and AVP (every other section receiving the TRPV4 and OXT combination). Therefore cell counts of TRPV4 and AVP labelled populations were obtained from alternate sections. As the effective size of these sections was 80 μm, no correction was made for double counting errors.

### 2.7 Antibody Specificity

**Figure 1:**
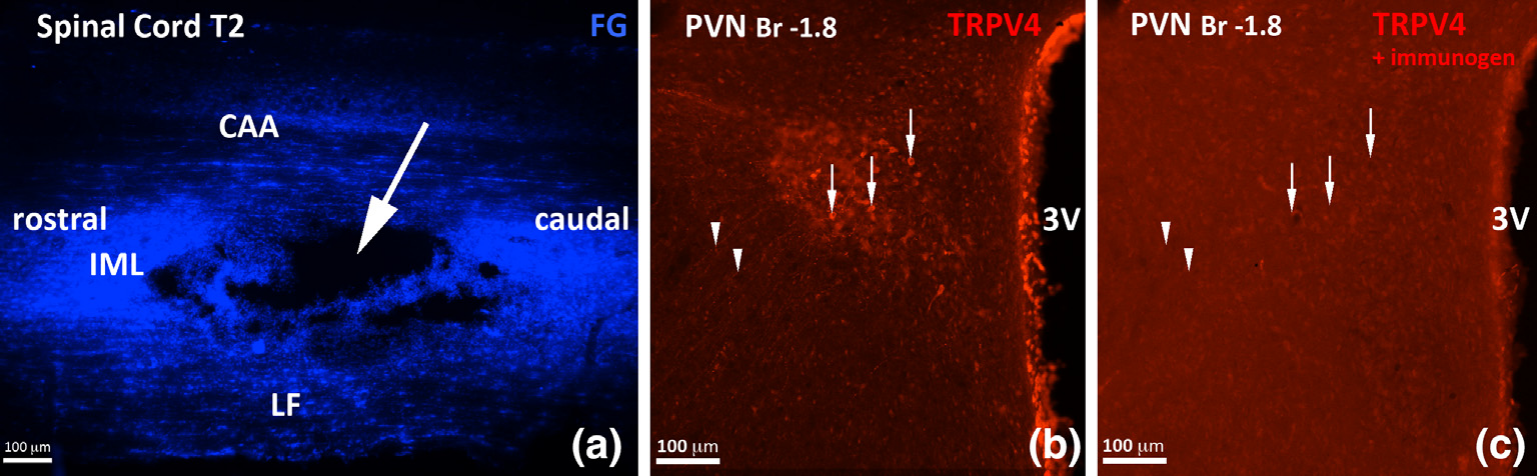
**Injection site and immunogen preadsorption control for anti‐TRPV4 antibody**. Panel a: Micrograph of a longitudinal section of spinal cord, showing the FG injection site (arrow) into the IML of the T2 segment that resulted in labelling of neurons in the PVN. Abbreviations: CAA‐ central autonomic area, IML‐ intermediolateral cell column, LF‐ lateral funiculus. Panels b and c: Prior incubation of the anti‐TRPV4 antibody with its immunising peptide (Abcam 230,486 1 mg/mL, 1:1 with antibody overnight at 4 °C) abolished labelling. b and c show consecutive sections of the PVN labelled with anti‐TRPV4 (b) and the same antibody following the blocking step (c). Cell bodies (arrows) and fibres (arrowheads) are clearly seen in b, but absent in c. Abbreviations: Br‐Bregma, 3 V‐ 3rd ventricle [Color figure can be viewed at wileyonlinelibrary.com]

These are all commercial antibodies subject to routine quality assurance (Table 1). Where positive results were obtained the pattern of reactivity was characteristic of that particular antibody with distinct cell populations consistently labelled by that antibody on repeat assays. There was an absence of labelling with secondary antibodies alone. For the anti‐TRPV4 antibody a further antigen preadsorption control was included. Prior incubation of the antibody with the immunising peptide (Abcam 230,486 1 mg/mL, 1:1 with antibody overnight at 4 °C) abolished labelling (Fig 1bc). Although preadsorption controls for the antibodies to OXT and AVP were not undertaken, both these antibodies have been used in previous, published studies to identify MNC's in rat PVN (Nedungadi & Cunningham, 2014; Reuss, Brauksiepe, Disque‐Kaiser, & Olivier, 2017).

**Table 1:** Primary antibodies

Antibody	Immunogen	Source	Concentration
TRPV4	Synthetic peptide corresponding to region 742–753 within internal amino acids 720–769. Blocking peptide sequence (identified by mass spectrometry) is shown in bold. (VDEVNWSHWNQNLGIINEDPGK**NETYQYYGFSHT**VGRLRRDRWSSVVPRV) of Human TRPV4 isoform 2 (NP_671737)	Abcam Cat# Ab94868, lot# GR276084; RRID:AB_10675981; rabbit, polyclonal	1:400
Oxytocin	Synthetic peptide H‐CYIQNCPLG‐NH_2_, (Disulphide bond)	Peninsula Laboratories, LLC (supplied by BMA Biomedicals) Cat# T‐5021.0050; RRID:AB_518526; guinea pig, polyclonal	1:1000
Vasopressin	Synthetic peptide H‐CYFQNCPDRG‐NH_2_, (Disulphide bond)	Peninsula Laboratories, LLC (supplied by BMA Biomedicals) Cat# T‐5048.0050; RRID:AB_518680; guinea pig, polyclonal	1:800

## 3 RESULTS

### 3.1 Spinally projecting preautonomic neurons

The injection site was identified and confirmed as being in the left intermediolateral cell column in all those animals whose tissues were used for immunohistochemistry (Figure 1a).

**Figure 2:**
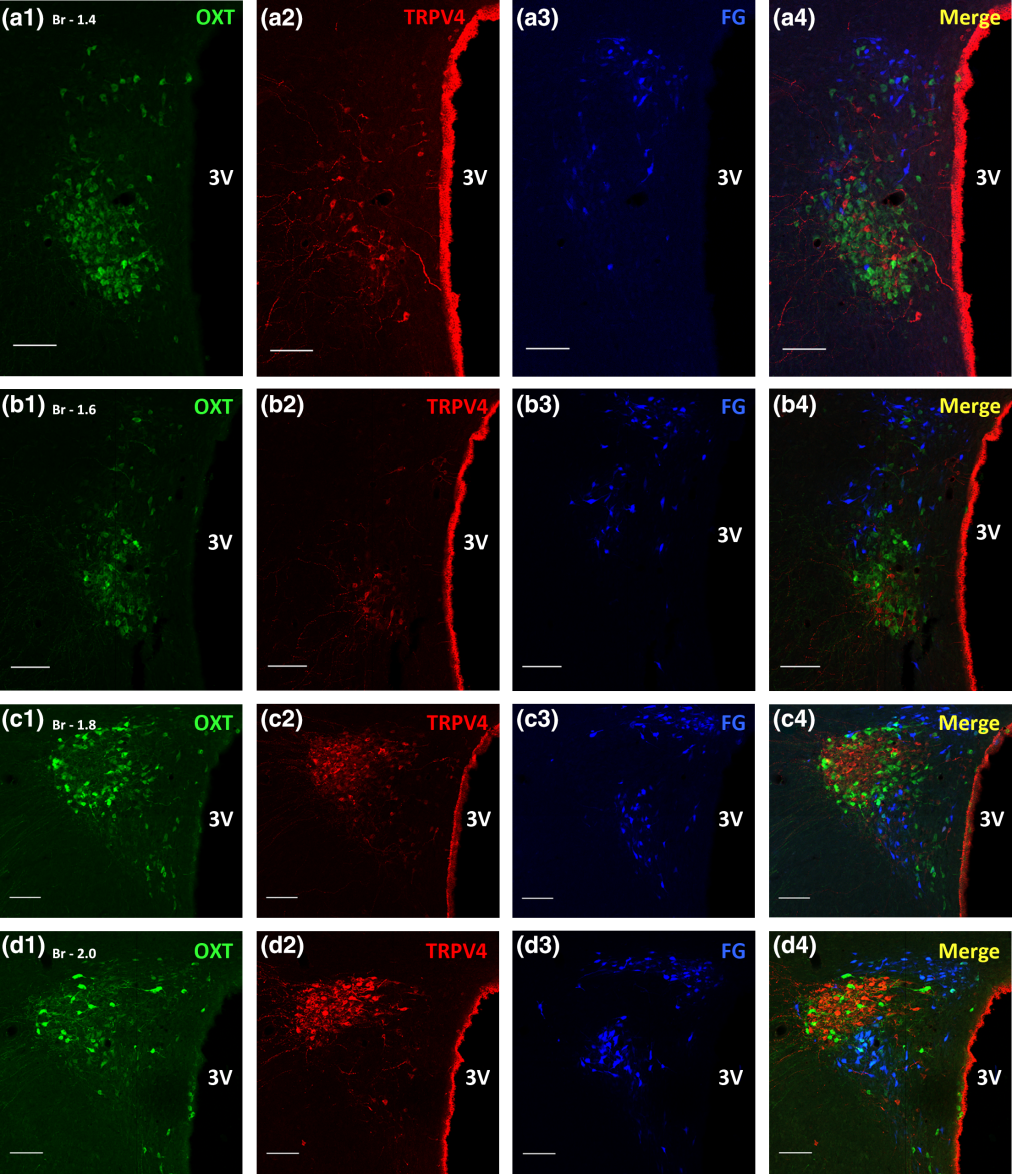
**Oxytocin immunoreactive‐, TRPV4 immunoreactive‐ and spinally projecting preautonomic neurons within the PVN.** The series a → d are PVN sections moving rostro‐caudally from Bregma −1.4 to −2.0. Panels a1‐d1 are oxytocin (OXT, green) neurons, a2‐d2 are transient receptor potential vanilloid 4 (TRPV4, red) neurons and a3‐d3 are spinally projecting preautonomic neurons retrogradely labelled with FluoroGold (FG, blue). Panels a4‐d4 are the merge of these three. All three cell groups occupy distinct regions of the PVN. Spinally projecting preautonomic neurons lie dorsal (panels a4, b4) and dorso‐ventral (panels c4, d4) to the mixed OXT and TRPV4 cells. Scale bar = 100 μm. Abbreviations: Br‐Bregma, 3 V‐ 3rd ventricle [Color figure can be viewed at wileyonlinelibrary.com]

Within the PVN, spinally projecting preautonomic neurons labelled with FG were ipsilateral to the spinal cord injection site although some neurons in the contralateral PVN were also evident. Spinally projecting preautonomic neurons were distributed rostro‐caudally (Bregma −1.40 to −2.12) within the parvocellular division of the PVN (Figures 2 & 4: a3‐d3). Within the more caudal regions of the PVN the spinally projecting neurons were confined to the dorsal and ventral regions of the nucleus with an intermediate “empty” region corresponding to the posterior magnocellular and medial parvocelluar regions (Figures 2 & 4: d3). The number of FG labelled cells on the ipsilateral side to the injection site was 896 ± 220 SD (n = 6), comparable to previous counts of spinally projecting preautonomic neurons within the PVN (Sawchenko & Kuypers 1980; Watkins, Cork & Pyner 2009).

### 3.2 TRPV4 ion channel expressing neurons

**Figure 3:**
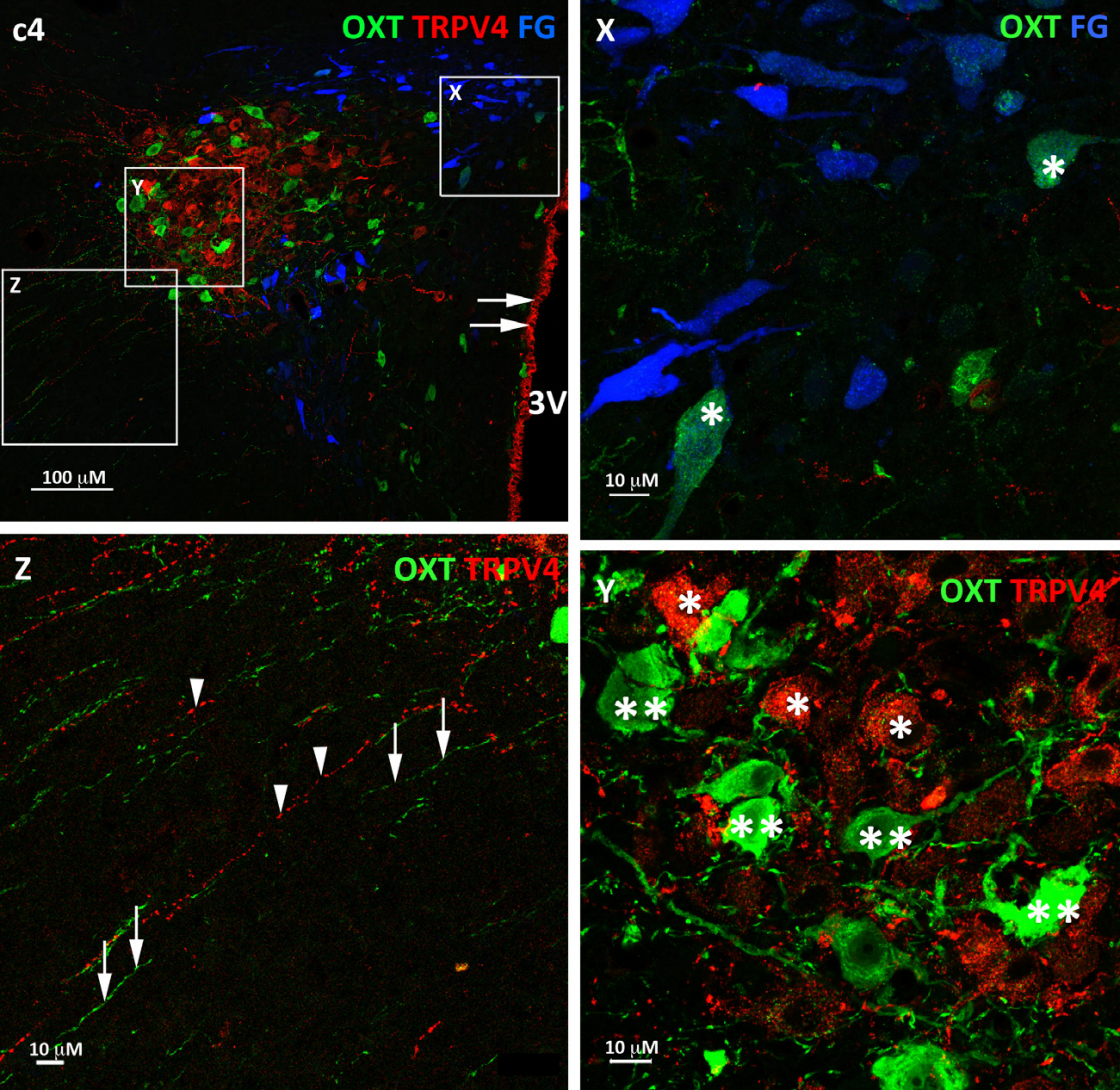
**Relationship between oxytocin immunoreactive‐ TRPV4 neurons and spinally projecting preautonomic neurons within the PVN.** Panel c4 from Figure 2 with high magnification insets (X, Y and Z) to show the relationship between the different cell groups: OXT/spinally projecting preautonomic neurons inset X, OXT/TRPV4 cells inset Y, OXT/TRPV4 fibres inset Z.Expanded inset X: Occasionally FG labelled spinally projecting preautonomic neurons also expressed OXT (*) Expanded inset Y: The OXT and TRPV4 cells are in close proximity with one another but OXT cells (*) are distinct from TRPV4 cells (**). Expanded inset Z: Similarly, the fibres (OXT fibres arrows, TRPV4 fibres arrowheads) extending laterally from the cells are separate from one another. Abbreviation: 3 V‐ 3rd ventricle [Color figure can be viewed at wileyonlinelibrary.com]

**Figure 4:**
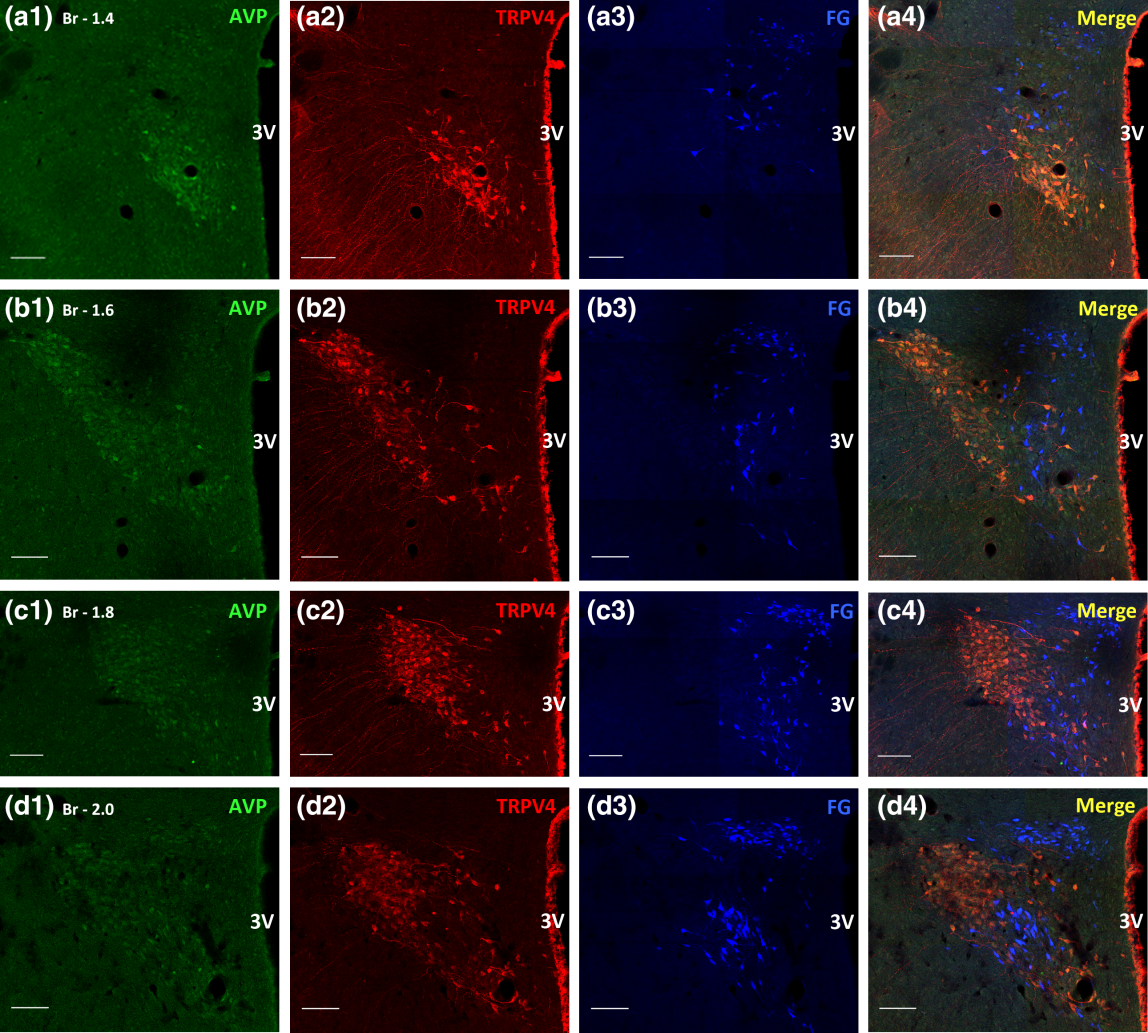
**Arginine‐vasopressin immunoreactive‐, TRPV4‐ immunoreactive and spinally projecting preautonomic neurons within the PVN.** The series a ‐ > d are PVN sections moving rostro‐caudally from Bregma −1.4 to −2.0. Panels a1‐d1 are arginine‐vasopressin (AVP, green) neurons, a2‐d2 are transient receptor potential vanilloid 4 (TRPV4, red) neurons and a3‐d3 are spinally projecting preautonomic neurons retrogradely labelled with FluoroGold (FG, blue). Panels a4‐d4 are the merge of these three. All of the TRPV4 labelled cells are vasopressinergic and appear orange/red in the merged panels. A small proportion of AVP cells do not express TRPV4 protein. The blue FG labelled cells lie predominantly medial (panel c4) and dorso‐ventral (panel d4) to the orange AVP/TRPV4 immunoreactive cell group. Scale bar = 100 μm. Abbreviations: Br‐Bregma, 3 V‐ 3rd ventricle [Color figure can be viewed at wileyonlinelibrary.com]

**Figure 5:**
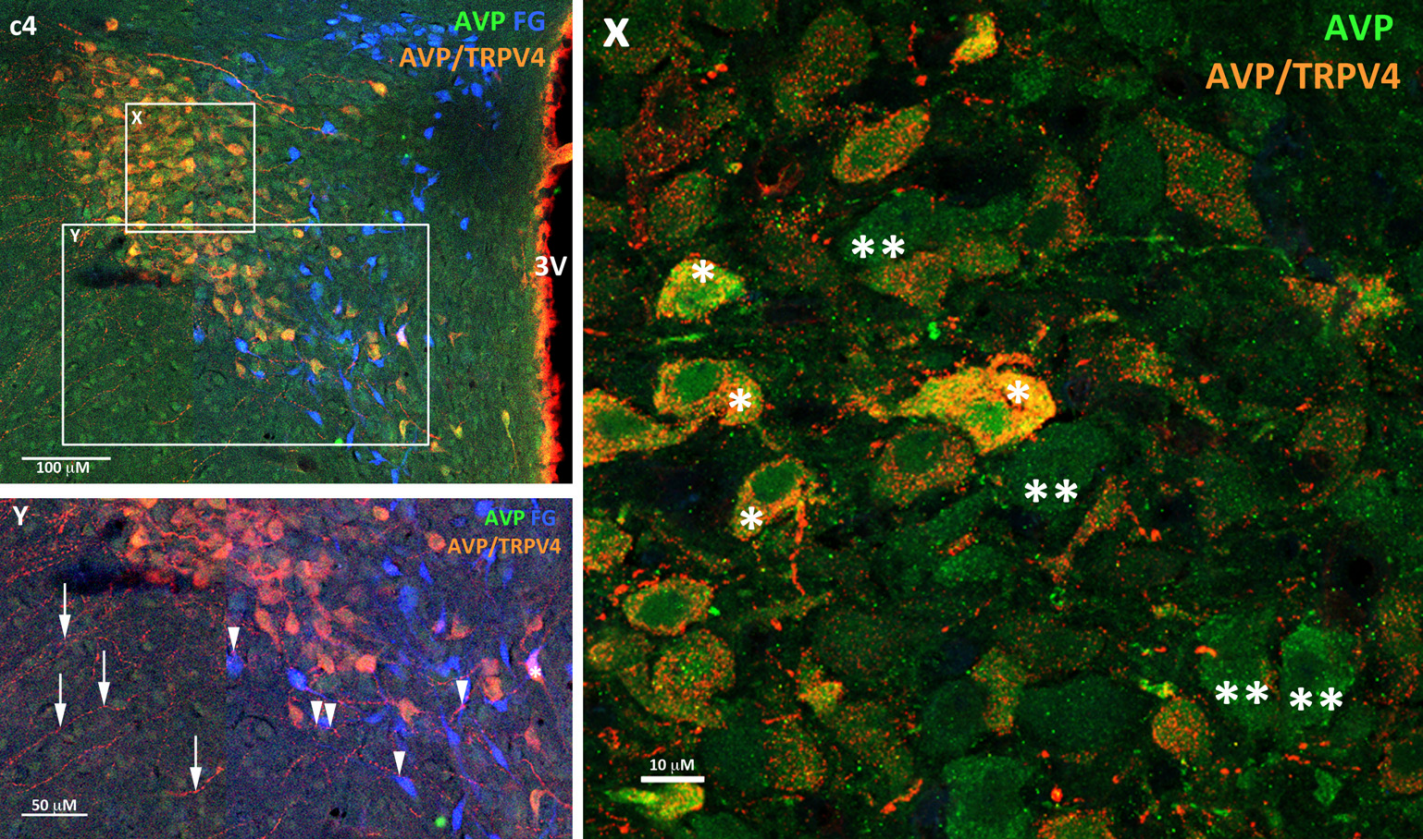
**Transient receptor potential vanilloid 4 is expressed exclusively on AVP neurons within the PVN.** Panel c4 from Figure 4 with high magnification insets to show that TRPV4 is present on the majority of vasopressinergic (AVP) cells (inset X) and spinally projecting preautonomic neurons are separate from these cell groups (inset Y). Expanded inset X: The majority of AVP neurons express TRPV4 (AVP/TRPV4*), however 37% of AVP neurons do not express TRPV4 (**). Expanded inset Y: A single spinally projecting preautonomic neuron immunoreactive for AVP and TRPV4 (*). This was the only occasion on which a triple labelled cell was seen. Otherwise spinally projecting preautonomic neuron (FG, blue) were always distinct from the AVP/TRPV4 (orange) and AVP (green) cells. Fine varicose fibres labelled with TRPV4 radiate laterally from the AVP/TRPV4 cells (arrows). On occasion these fibres are closely apposed to the spinally projecting preautonomic neurons (arrowheads). Abbreviation: 3 V‐ 3rd ventricle [Color figure can be viewed at wileyonlinelibrary.com]

The majority of TRPV4 labelled cells were prevalent in the posterior magnocellular region of the PVN (Figures 2 & 4: a2‐d2). The TRPV4 labelling appeared granular and was evident throughout the cytosol (Figure 5: c4, Y, red cells labelled with double asterix). Immunoreactivity for TRPV4 was evident on neurons in the SON (not shown) and also those lining the 3rd ventricle (Figure 5: c4 arrows). Fine varicose fibres identified with TRPV4 immunoreactivity coursed laterally and ventrally between the PVN and SON (Figure 3: c4, Z) but no fibres were observed to emanate from the TRPV4 cells lining the 3rd ventricle. A population of TRPV4 cells was also observed dorsal to the fornix with fibres extending laterally and a further population ventral to the spinally projecting preautonomic neurons with fibres projecting towards the SON. In the most caudal region of the PVN lateral to and just beyond the “wings” of the spinally projecting preautonomic neuronal grouping a few neurons were found to express TRPV4 and again fibres extended towards the supraoptic decussation and optic tract (not shown).

### 3.3 Relationship between TRPV4 and spinally projecting neurons

Rarely were neurons expressing TRPV4 identified as spinally projecting preautonomic neurons apart from in one case found in the caudal region of the PVN and this neuron was also vasopressinergic (Figure 5: c4, Y asterix). Spinally projecting preautonomic neurons and TRPV4 neurons were observed as discrete populations throughout the rostral‐caudal extent of the PVN nucleus (Figures 2, 3, 4, 5). In the posterior magnocellular region where TRPV4 immunoreactive cells were most abundant, the spinally projecting neurons were positioned immediately dorsal and ventral to the TRPV4 cells with some overlap between the distinct populations.

### 3.4 Relationship between TRPV4 and vasopressinergic neurons

Neurons immunoreactive for vasopressin were predominantly confined to the posterior magnocellular region with a posterior dorsolateral distribution (Figure 4: a1‐d1). The number of vasopressinergic neurons counted was 1,065 ± 191 SD (n = 4), consistent with previous studies (Rhodes, Morrell & Pfaff, 1981; Sawchenko & Swanson, 1982). This region also contained the majority of the TRPV4 immunoreactive neurons (Figures 2 & 4: a2‐d2). All TRPV4 neurons were found to be vasopressinergic (Figure 5: c4, X) with TRPV4 expressing neurons making up 63% of the total vasopressinergic neuronal population. A further TRPV4‐AVP expressing population was also observed within the SON (not shown). The only neuron identified as a spinally projecting neuron and expressing TRPV4, also contained vasopressin immunoreactivity (Figure 5: Y asterix, see section 3.3).

### 3.5 Relationship between TRPV4 and oxytocinergic neurons

Oxytocinergic neurons were present rostro‐caudally throughout the PVN and were most abundant in the posterior magnocellular cell region (Figure 2: a1‐d1). The TRPV4 neurons and fibres occupied the same region but the two populations were separate and there was no evidence of TRPV4 being expressed on oxytocinergic neurons (Figure 3: Y double asterix OXT neurons, single asterix TRPV4 neurons). A similar pattern was observed for the SON i.e. intermingled but discrete TRPV4 and oxytocinergic pools of neurons (not shown).

### 3.6 Relationship between spinally projecting‐ and vasopressinergic neurons and spinally projecting‐ and oxytocinergic neurons

Only occasionally were the spinally projecting neurons found to be vasopressinergic (Figure 5: Y asterix). A similar pattern for OXT was observed, however while only a small number of spinally projecting preautonomic neurons were immunoreactive for OXT it was more than for AVP (Figure 3: X asterix). The spinally projecting –oxytocinergic neurons were predominantly located within the caudal parvocellular regions of the PVN (Figure 3: X asterix) although a few were found in the posterior magnocellular region, the region where TRPV4 expressing neurons were most abundant.

## 4 DISCUSSION

This study investigated the anatomical relationship existing between the TRPV4 ion channel and neurons known to be involved in modulating osmotic stress and sympathetic output from the PVN. The study has provided evidence that the TRPV4 ion channel was localised within the posterior magnocellular subdivision of the PVN. This region also contained both the magnosecretory neurons containing OXT and AVP, in agreement with previous studies (Swanson & Sawchenko, 1983; Son et al., 2013). Neurons immunoreactive for TRPV4 were exclusively vasopressinergic and TRPV4 had no relationship with the oxytocinergic population i.e. not co‐localised. In addition, spinally projecting preautonomic neurons within the parvocellular cell group of the PVN did not show any anatomical relationship with the TRPV4 ion channel. The TRPV4 channel was also expressed within the SON, where again TRPV4 was always associated with AVP immunoreactive neurons.

### 4.1 TRPV4 ion channel

The TRPV4 ion channel, a member of the transient receptor potential family of cation channels, responds to a broad range of stimuli including osmoregulation (Liedtke et al., 2000; Voets, Talavera, Owsianik, & Nilius 2005; Stiber, Tang, Li, & Rosenberg 2012). In addition to showing the colocalisation of TRPV4 with AVP in the posterior magnocellular subdivision of the PVN we also found varicose fibres immunoreactive for TRPV4 extending laterally and ventrally. The projections are assumed to be vasopressinergic since such fibres were seen originating from AVP neurons but co‐localisation with TRPV4 could not be established. It is not possible to unequivocally determine whether these fibres are axons or dendrites. Previous studies have shown vasopressinergic neurons within the magnocellular subdivision of the PVN extend varicose fibres (axons) laterally and ventrally towards the median eminence and SON while the dendrites spread into the parvocellular region (Son et al., 2013). In contrast, Carreño and colleagues described TRPV4 to be on both parvocellular and magnocellular neurons of the PVN colocalised with AVP, with a similar pattern for the SON (Carreño, Ji, & Cunningham, 2009). However, from their published paper it is not possible to unequivocally identify the TRPV4 immunoreactive neurons as parvocellular. We have taken serial sections throughout the rostrocaudal extent of the PVN and our high power confocal images unambiguoulsy demonstrate TRPV4 expression on vasopressinergic MNCs. Our images clearly show granular expression of TRPV4 protein throughout the cytosol (Figure 4: c4, Y, red cells labelled with double asterix) and on fine varicose fibres extending laterally and ventrally between the PVN and SON (Figure 4: c4, Z). Our observations are in agreement with recent work showing TRPV4 expressed throughout the cytosol, dendrites and axons of cultured hippocampal neurons (Gu et al., 2017). While the channel may be found throughout the cytoplasm in unstimulated cells, it is likely that mechanical perturbation leads to recruitment of intracellular pools of TRPV4 to the plasma membrane as has been shown in native and recombinant TRPV4‐expressing cells (Baratchi et al., 2016). The granular nature of our staining is of interest. It may be accounted for by co‐localisation of TRPV4 with actin and microtubule rich structures, which can resemble focal adhesion points (Goswami, Kuhn, Heppenstall, & Hucho, 2010). Certainly the expression of TRPV4 appears to be highly dynamic: in non‐stimulated endothelial cells the channel has been found clustered in small protein islands, subsequent shear stress generated by blood flow leads to the formation of smaller clusters with the majority of the TRPV4 protein then located outside of these (Baratchi, Knoerzer, Khoshmanesh, Mitchell, & McIntyre, 2017). The TRPV4 channel appears to function as part of a complex containing cytoskeletal proteins and regulatory kinases, where it can integrate intracellular signalling with cytoskeletal dynamics (Goswami et al., 2010).

Spinally projecting preautonomic neurons in the parvocellular region of the PVN did not express TRPV4 or contain AVP and a topgraphical segregation was evident between the spinally projecting and the TRPV4‐AVP neurons. Topgraphical segregation between AVP and PVN‐RVLM projecting neurons has also been reported (Son et al., 2013). Only one preautonomic neuron was found to be immunoreactive for both TRPV4 and AVP. *In situ* hybridisation suggests that 40% of spinally projecting neurons contain mRNA for AVP. In colchicine treated rats at least 35% of the PVN‐spinally projecting neurons contained AVP and sparsely distributed AVP axons can be found apposed to adrenal medullary and stellate ganglion projecting sympathetic preganglionic neurons in the spinal cord (Cechetto & Saper, 1988; Ranson, Motawei, Pyner, & Coote, 1998; Motawei et al., 1999; Hallbeck, Larhammar, & Blomqvist, 2001). Functionallly, stimulation of the PVN‐spinal projection leads to a vasopressin‐dependent excitation of sympathetic preganglionic neurons in the spinal cord of the rat via a V1a receptor (Gilbey, Coote, Fleetwood‐Walker, & Peterson, 1982; Backman & Henry, 1984; Ma & Dun, 1985; Malpas & Coote, 1994; Sermasi, Howl, Wheatly, & Coote, 1998). The fact that rarely do PVN parvocellular neurons contain AVP seems at odds with the functional evidence and it may reflect that within the parvocellular neurons AVP is stored as neurophysin (Swanson, 1977; White, Krause, & McKelvy, 1986).

Putative dendrites from the TRPV4‐AVP neurons were seen to project into and through the preautonomic neurons and closley appose them. Again a similar pattern has been reported for PVN‐RVLM projecting neurons (Son et al., 2013). As all TRPV4 neurons are vasopressinergic and these neurons are a separate population to the parvocellular neurons irrespective of autonomic target, then we can suggest that the PVN‐RVLM would not express TRPV4. Indeed a recent study provides evidence for another TRP family member TRPM4 to be expressed on PVN‐RVLM preautonomic neurons (Son et al., 2013).

Oxytocinergic MNCs had a similar distrubution within the posterior magncoellular region of the PVN as vasopressinergic MNCs. However, the OXT neurons and fibres did not express TRPV4. Like vasopressin around 40% of spinally projecting neurons contain OXT mRNA (Hallbeck, Larhammar, & Blomqvist, 2001) while for those parvocellular neurons projecting to the stellate ganglion about 10% have been shown to be oxytocinergic (Jansen, Wessendorf, & Loewy, 1995). Functionally, OXT has been implicated in autonomic regulation with a direct action on sympathetic preganglionic neurons (Gilbey, Coote, Fleetwood‐Walker, & Peterson, 1982; Yasphal, Gauthier, & Henry, 1987; Sermas & Coote, 1994; Deusaules, Reiter, & Feltz, 1995; Yang, Wheatley & Coote, 2002). While the primary roles of AVP and OXT are very different, oxytocinergic MNCs can also be activated by increasing osmolality but our evidence would indicate the TRPV4 ion channel is not part of the mechanism (Leng et al., 2001; Oliveria et al., 2004).

### 4.2 TRPV4‐parvocellular interaction

The paraventricular nucleus of the hypothalamus has been shown to be critical to sensing and responding to changes in plasma osmolality (Bourque, 2008). Disturbances in osmolality and the evoked cellular response involve TRPV4‐activation coupled to the low‐conductance calcium‐activated potassium (SK) channel. An *in vitro* study using mouse brain slices and rat isolated PVN neurons demonstrated that anatomically and morphologically defined parvocellular neurons responded to osmolality (Feetham, Nunn, Lewis, Dart, & Barrett‐Jolley, 2015b). Superfusion of the brain slices with hypotonic artificial cerebrospinal fluid was found to reduce action current frequency and these effects were mediated by coupling of TRPV4/SK channels. Similarly, an *in vivo* study investigated whether hypotonic TRPV4 driven neuronal inhibition modulated cardiovascular parameters. In mice, intracerebroventricular administration of hypotonic solutions decreased mean blood pressure but not heart rate and inhibition of the TRPV4 channels abolished these effects (Feetham, Nunn & Barrett‐Jolley, 2015a). These studies support a central TRPV4 channel as important for sensing osmolality and the authors proposed the effects of its activation to be mediated by the channel expressed on spinally projecting preautonomic neurons (Feetham, Nunn, Lewis, Dart, & Barrett‐Jolley, 2015b). However, we have shown the TRPV4 channel is only associated with AVP MNCs. Therefore we suggest that the spinally projecting preautonomic neurons are activated indirectly by AVP released from MNCs in the same vicinity, this alternative explanation would be compatible with both our observations and the functional studies of Feetham and colleagues.

Release of AVP from MNCs is closely related to electrical activity of these cells (Leng, Brown, & Russell, 1999). Magnocellular neurosecretory cells appear to positively correlate their rate of action potential discharge with extracellular fluid osmolality (Bourque, 1998). Under normal body fluid osmolality, the firing rate of the MNCs (~1–3 Hz) mediates basal AVP secretion, whereas hypotonicity and hypertonicity decrease and increase respectively, firing frequency and AVP release (Bourque, 1998).

Recently crosstalk between AVP MNCs and PVN‐RVLM projecting preautonomic neurons has been proposed (Son et al., 2013). Activity dependent dendritic release of AVP from neurosecretory neurons has been shown to stimulate PVN‐RVLM preautonomic neurons demonstrating a mechanism for interpopulation crosstalk (Son et al., 2013). The released AVP is proposed to act as a diffusible signal between populations of neurons within the PVN. A central osmotic challenge administered via intracarotid infusion of increasing concentrations of sodium chloride results in concentration dependent increases in renal sympathetic nerve activity (Chen & Toney, 2001). This effect was shown to be due to intranuclear release of AVP from MNCs because blockade of the V1a receptor within the PVN blunted the increase in renal sympathetic activity suggesting the AVP was contributing to the sympathoexcitation (Son et al., 2013). Thus central osmotic challenge is able to modulate sympathetic output; and separate studies have shown that this can be attenuated centrally either by TRPV4 inhibition (Feetham, Nunn & Barrett‐Jolley, 2015a) or blockade of V1a receptors on spinally projecting preautonomic neurons (Son et al., 2013). Our demonstration of TRPV4 on AVP MNCs suggests these effects may be mediated via release of AVP from these neurons onto preautonomic sympathetic neurons with which they are in close proximity.

### 4.3 Conclusion

Our study provides an anatomical understanding of how changes in osmolality may affect sympathetic output from spinally projecting neurons within the PVN of the hypothalamus. Osmotic sensing by the TRPV4 ion channel expressed on AVP MNCs may lead to dendritic release of AVP and its subsequent diffusion onto preautonomic networks. The precise mechanism for sensing and signalling of osmotic disturbances and thus blood plasma volume has important implications in heart failure as recent evidence would suggest AVP modulation of sympathoexcitibility is impaired in disease. A reduction in the expression of SK (small conductance K_ca_ channels) in hypothalamic MNCs in heart failure rats has been shown to contribute to the hyper‐excitibility of those neurons (Ferreira‐Neto, Biancardi & Stern, 2017). An increase in hypothalamic MNC excitability could lead to increased AVP release and the well documented sympathoexcitation observed in heart failure animals (Abboud, 2010). Functional studies in combination with anatomical analyses detailing the location of osmosensitive proteins on cell groups within cardiovascular control centres and their interconnections, is gradually revealing the mechanisms underpinning cardiovascular homeostasis.

## CONFLICT OF INTEREST STATEMENT

The authors declare they have no conflict of interest.

## ACKNOWLEDGMENTS

This work was supported by a BHF project grant (PG/14/53/309000) awarded to SP.

We thank Ms Joanne Robson for her expert contribution to the confocal imaging, Ms Demi Minhinnett for assistance with animal work and Dr Adrian Brown for his expertise in Mass Spectrometry (MALDI‐TOF MS).

In memory of Professor John H Coote (1936‐2017).
